# Slow and temperature‐mediated pathogen adaptation to a nonspecific fungicide in agricultural ecosystem

**DOI:** 10.1111/eva.12526

**Published:** 2017-09-14

**Authors:** Meng‐Han He, Dong‐Liang Li, Wen Zhu, E‐Jiao Wu, Li‐Na Yang, Yan‐Ping Wang, Abdul Waheed, Jiasui Zhan

**Affiliations:** ^1^ State Key Laboratory of Ecological Pest Control for Fujian and Taiwan Crops Fujian Agriculture and Forestry University Fuzhou China; ^2^ Fujian Key Laboratory of Plant Virology, Institute of Plant Virology Fujian Agriculture and Forestry University Fuzhou China; ^3^ Key Lab for Biopesticide and Chemical Biology Ministry of Education Fujian Agriculture and Forestry University Fuzhou China

**Keywords:** *Alternaria alternata*, epistasis, evolution of antimicrobial resistance, negative pleiotropy, population genetics

## Abstract

The spread of antimicrobial resistance and global change in air temperature represent two major phenomena that are exerting a disastrous impact on natural and social issues but investigation of the interaction between these phenomena in an evolutionary context is limited. In this study, a statistical genetic approach was used to investigate the evolution of antimicrobial resistance in agricultural ecosystem and its association with local air temperature, precipitation, and UV radiation. We found no resistance to mancozeb, a nonspecific fungicide widely used in agriculture for more than half a century, in 215 *Alternaria alternata* isolates sampled from geographic locations along a climatic gradient and cropping system representing diverse ecotypes in China, consistent with low resistance risk in many nonspecific fungicides. Genetic variance accounts for ~35% of phenotypic variation, while genotype–environment interaction is negligible, suggesting that heritability plays a more important role in the evolution of resistance to mancozeb in plant pathogens than phenotypic plasticity. We also found that tolerance to mancozeb in agricultural ecosystem is under constraining selection and significantly associated with local air temperature, possibly resulting from a pleiotropic effect of resistance with thermal and other ecological adaptations. The implication of these results for fungicide and other antimicrobial management in the context of global warming is discussed.

## 1 INTRODUCTION

Antimicrobial resistance, the ability of pathogens to withstand the action of pesticides and antibiotic drugs, has recently been rated to have a disastrous impact on a wide array of natural and social issues such as public and animal health, food security, socioeconomic development, and ecological sustainability to an extent similar to those caused by global climate change (Gelband & Laxminarayan, 2015; Gould, 2010; Hiltunen, Virta, & Laine, 2017) such as increases in mean and fluctuation of world temperature (IPCC 2007). Depending on their action modes, antimicrobials can be classified as site‐specific and site‐nonspecific, and genetic resistance to antimicrobials can emerge either from a sequential accumulation of multiple amino acid substitutions in many independent genes of pathogen genomes such as resistance to cyproconazole in sterol demethylation inhibitor group of site‐nonspecific antimicrobials (Mohd‐Assaad, McDonald, & Croll, 2016; Zhan, Stefanato, & McDonald, 2006) or from a single point mutation such as resistance to metalaxyl, phenylamide, and benzimidazole (Chen et al., 2009; Gisi et al., 2000) in many site‐specific antimicrobials. Consequently, it is commonly believed that the risk of developing resistance to site‐nonspecific antimicrobials is lower than to site‐specific antimicrobials. Evolutionary theory also hypothesizes that resistant mutants are selectively advantageous in the presence of antimicrobials but carry a fitness penalty in the absence of antimicrobials because mutations often impede important cellular and biochemical functions (Cooper, Ostrowski, & Travisano, 2007; Hall, Angst, Schiessl, & Ackermann, 2015), leading to resistance polymorphisms in natural pathogen populations.

In addition to action modes and genetic characters of pathogen mutants in the action sites, many other genetic, environmental, and social factors can influence the population dynamics and evolution of antimicrobial resistance in pathogens. Mixtures and rotations of antimicrobials with different action modes delay the development of pathogen resistance due to the widened action spectrum (Valencia‐Botín, Jeffers, Palmer, & Buck, 2013) and reduced selection pressure (Hobbelen, Paveley, Oliver, & Van den Bosch, 2013; Perron, Inglis, Pennings, & Cobey, 2015). Negative pleiotropy and interactions with other parts of the pathogen genome may modify the impact of a mutation in the action sites or shift its effect from beneficial to neutral or even deleterious. Such background‐dependent benefits or costs of mutations to antimicrobial resistance resulting from genetic epistasis (Schenk, Szendro, Salverda, Krug, & de Visser, 2013; Vogwill, Kojadinovic, & MacLean, 2016) and trade‐offs (Ferenci, 2003; Schenk et al., 2015) have recently been documented in several antimicrobial–pathogen systems. Furthermore, as an omnipresent environmental factor governing biotic and abiotic processes at all levels, temperature is expected to play an important and multifaceted role in the evolution of antimicrobial resistance through its impact, direct and/or indirect, on chemical features of antimicrobial compounds; genetic, biological, physiological, and evolutionary characters of pathogens; and the interaction between antimicrobials and pathogens. Indeed, previous studies revealed that temperature regulated the toxicity of molecular compounds (Khan & Akram, 2014), expression of functional genes (McGann, Ivanek, Wiedmann, & Boor, 2007), trade‐off of ecological traits (Handel, Lebarbenchon, Stallknecht, & Rohani, 2014; Yang et al., 2016), and generation and maintenance of antimicrobial resistance (Rodríguez‐Verdugo, Gaut, & Tenaillon, 2013).

Fungicides are a group of antimicrobials widely used to manage fungal diseases in agricultural system. Evolution of fungicide resistance in pathogen populations greatly threatens food security and ecological sustainability. Understating how resistance evolves in pathogen populations and how biotic and abiotic factors affect the evolution of resistance is critical to effectively manage fungicides and other antimicrobials such as antibiotic drugs in medicines. Mancozeb is a broad‐spectrum fungicide with a multisite protective action (Gullino et al., 2010). It inhibits or interrupts at least six different biochemical processes within the cell cytoplasm and mitochondria, resulting in inhibition of spore germination in fungi due to its disruption of lipid metabolism, respiration, and production of adenosine triphosphate (Iorio et al., 2015; Santos, Simoes, & Sa‐Correia, 2009). Since its introduction in 1962, this fungicide has been widely used to control more than 400 plant diseases over 70 different hosts including field crops, fruits, nuts, vegetables, and ornamentals in almost 120 countries (Gullino et al., 2010). Low risk of resistance development in pathogen populations is believed to be one of a number of key attributes contributing to the dominant position of mancozeb in global agrochemical markets (Gullino et al., 2010). Despite its intensive application over the 50 years since its commercialization, there has been no documented occurrence of resistance to mancozeb in natural fungal populations although substantial variation in baseline sensitivity and tolerance has been documented in pathogen populations among locations and species (Gullino et al., 2010; Torres‐Calzada et al., 2015).

Mancozeb has been routinely used to control potato early blight (Gent & Schwartz, 2003; Malandrakis, Apostolidou, Markoglou, & Flouri, 2015), a foliar disease that forms dark brown‐colored spots on leaves that are necrotic in the center with a halo‐like pattern of concentric rings. The disease is found worldwide and can cause significant damage to plants when environmental conditions are conducive particularly in warm and alternating dry and high humidity periods (Leiminger & Hausladen, 2012). Although potato early blight can be caused by both *A. alternata* and *A. solani* (Boiteux & Reifschneider, 1994; Leiminger & Hausladen, 2012; Zheng, Zhao, Wang, & Wu, 2015), the former has emerged as the main causal agent in China (Meng et al., 2015b). The two species can be distinguished from each other by spore characters and PCR amplifications of ITS and other housekeeping genes (Wier, Huff, Christ, & Romaine, 1998; Zheng et al., 2015). *Alternaria alternata* produces conidia containing 8–12 spores in length with numerous secondary and occasionally tertiary chains branching from apical and median cells, while *A. solani* produces conidia having 9–11 transverse septa and 1–2 longitudinal septa with one long to ovoid beak (Wier et al., 1998; Zheng et al., 2015). In addition to potato early blight, *A. alternata* can also cause leaf spots, blight, and other diseases on numerous plant species (Wier et al., 1998; Woudenberg, Groenewald, Binder, & Crous, 2013) although host specificity has been documented in some studies (Elena, 2006; Woudenberg et al., 2015). The pathogen has a global distribution and is dispersed by rain‐splash, wind, and on infected plant material (Reis et al., 2006). No teleomorphs (sexual fruiting body) have yet been observed either in the laboratory or in field, but cryptic sexual reproduction has been documented by molecular analyses of genetic variation and phylogenetic trees (Meng et al., 2015a), providing the pathogen an additional mechanism beyond mutation alone to generate genetic variation for ecological adaptation including the development of fungicide resistance. Field epidemics are believed to be initiated by primary inoculum originating from conidiospores (Rotem, 1994).

The impacts of antimicrobial resistance and climate change on nature and human society have typically been investigated independently (Tenover, 2006; Vittoz et al., 2013). A synergistic analysis of interactions between climatic factors and antimicrobial resistance in an evolutionary context has been rarely conducted but fundamental to address the growing public concerns of human and animal health, food security, and ecological sustainability under changing environments. In this study, we used the mancozeb–*A. alternata* interaction as a model to test the hypotheses that local temperature, precipitation, and UV radiation play an important role in the antimicrobial evolution of pathogens. Through statistical genetic analysis of molecular and physiological markers, the specific objectives of the present study were to (i) determine the spatial distribution of mancozeb tolerance in pathogen populations collected from various geographic locations representing various ecotypes and climatic zones; (ii) investigate the relative contribution of genetic variance and phenotypic plasticity to the development of resistance in mancozeb; and (iii) evaluate the effect of local temperature, precipitation, and UV radiation on the evolution of mancozeb resistance.

## 2 MATERIALS AND METHODS

### 2.1 *Alternaria alternata* collections, DNA extraction, and SSR assays

**Figure 1:**
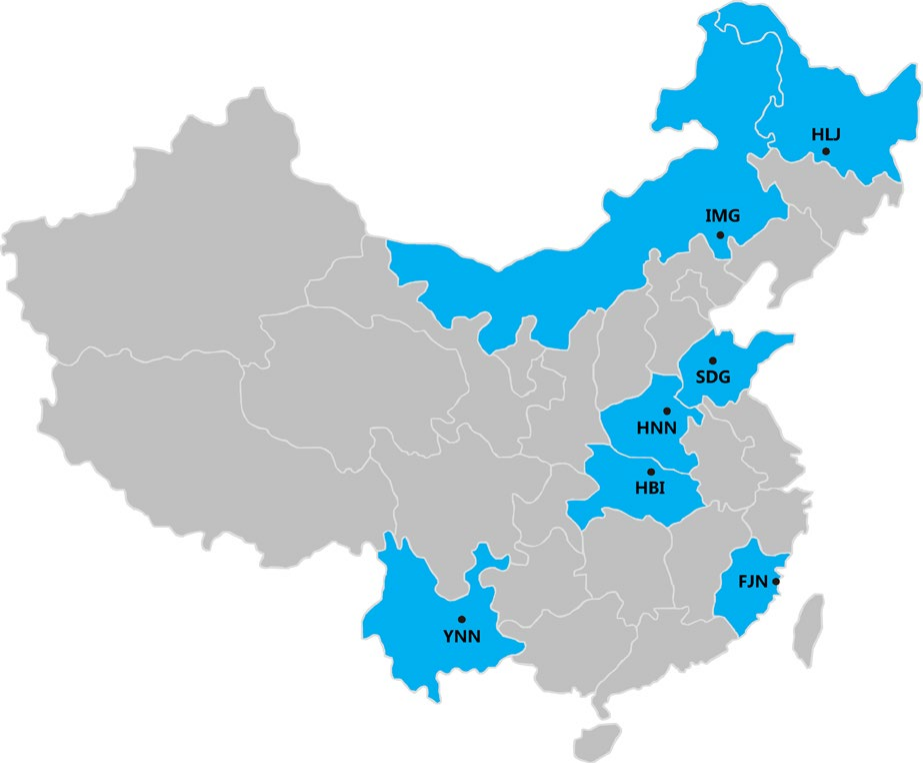
Map showing the geographic locations of the seven *Alternaria alternata* populations included in this study

*Alternaria alternata* isolates were collected from seven potato fields located in Fujian (FJN), Heilongjiang (HLJ), Henan (HNN), Hubei (HBI), Inner Mongolia (IMG), Shandong (SDG), and Yunnan (YNN) provinces (Figure 1) during the 2011 and 2012 growing seasons and altitude of the location was recorded during collection. These locations were chosen to represent a climatic gradient covering the main potato production areas in the country. The pathogen isolates were previously genotyped with eight pairs of microsatellite markers and the detailed information on collection, isolation, DNA extraction, and microsatellite genotyping of the isolates can be found in these publications (Meng et al., 2015a,b). Briefly, potato leaves with typical early blight symptoms were sampled from plants at 1‐ to 2‐meter intervals and each infected leaf was packed separately in a sealed sandwich bag. The leaf samples were shipped to the laboratory for *A. alternata* isolation within 48 hr. To isolate the pathogen, infected leaves were first rinsed briefly with distilled water, surface‐sterilized with 75% alcohol for one minute, and then incubated at 24°C on 1% water–agar medium. After 24 hr, a single conidium was taken from each infected leaf, incubated on potato dextrose agar (PDA, potato 200 g/L, glucose 20 g/L, agar 20 g/L) plates, and then maintained for two weeks in an incubator set to 24°C under a dark condition (Meng et al., 2015a). The resultant isolates were purified three times by repeatedly transferring a single conidium to fresh PDA plates. Purified isolates were put into silica gel for long‐term storage. After the third round of purification, mycelia were harvested for DNA extraction using a plant gDNA kit (Promega Biotech. co. LTD., Beijing). Because potato early blight can be induced by pathogen complex, all isolates were checked morphologically by spore characterization under a light microscope (Meng et al., 2015a,b) and molecularly by PCR amplifications of ITS regions with primers ITS1 (5′‐TCCGTAGGTGAACCTGCGG‐3′) and ITS4 (5′‐TCCTCCGCTTATTGATATGC‐3′) and histone 3 gene with primers H3‐1a (5′‐ACTAAGCAGACCGCCCGCAGG‐3′) and H3‐1b (5′‐GCGGGCGAGCTGGATGTCCTT‐3′) using the protocols described previously (Meng et al., 2015a; Zheng et al., 2015).

Genomic DNA of the isolates were amplified with eight pairs of primers (*PAS1, PAS2, PAS3, PAS4, PAS5, PAS6, PAS7*, and *Ad8*) developed previously (Benichou, Dongo, Henni, Peltier, & Simoneau, 2009; Meng et al., 2015a). SSR amplification was executed in a total reaction volume of 25 μl containing 1 μl of *A. alternata* genomic DNA, 12.5 μl of 2X EasyTaq PCR SuperMix (‐dye) (Transgen Biotech Co., Ltd.), 9.5 μl of sterile water, and 1 μl each of forward and reverse primers. The primers were synthesized by Ruiboxingke Biotech Co. (Beijing) and labeled with different fluorescent dyes at the 5′ end. The programmer used for PCR was as follows: initially held at 95°C for 5 min; followed by 35 cycles of 94°C for 30 s, 57°C for 30 s (same annealing temperature for all primers), 72°C for 30 s; and then ended with an extension step of 72°C for 5 min. Amplicon sizes were determined by Ruiboxingke Biotechnology Co. Ltd using an ABI 3730XL automated DNA sequencer (Applied Biosystems) in which a DNA size ladder was included in each sample. Alleles were assigned based on the sizes of PCR amplicon that were generated by each pair of SSR primers using GeneMarker software version 1.31 with a binning procedure. PCR amplicons with an identical size generated by the same pair of primers were considered as an allele. Multilocus genotype for each isolate was formed by joining the alleles at each SSR locus in the same order across the eight primers, and GenClone 2.0 (Arnaud‐haond & Belkhir 2007) was used to determine whether isolates with the same multilocus genotypes were the asexual progeny of a genotype.

### 2.2 Measurement of mancozeb tolerance

A total of 215 *Alternaria alternata* isolates, each with a distinct genotype, were chosen for the fungicide experiment. The fungal isolates retrieved from long‐term storage were revived on PDA plates for six days. Mycelial plugs (ϕ = 5 mm) were transferred to fresh PDA plates either amended with 4 μg/ml, 10 μg/ml, and 18 μg/ml of mancozeb prepared from technical grade or without the fungicide in 9‐cm petri dishes. Our exploratory experiments showed that these concentrations provided the best resolution with the least experimental error. Many isolates did not grow when we used higher concentrations, while growth rates of many isolates did not change when we used lower concentrations. The plates were divided into three separate batches each corresponding to one of the three mancozeb concentrations selected and laid out in a completely randomized design using three replicates as recommended by previous studies (Zhan & McDonald, 2011; Zhan et al., 2006). Controls (without supplementation of mancozeb) were included in each batch of plates. Media, inoculations, and colony measurements for the entire experiment were made by the same person with all isolate–replicate combinations for a single fungicide concentration being assessed on the same day in a single incubator set to 24°C. Colonies were photographed daily between day two and day six postinoculation, and colony areas were measured with the image analysis software Assess (Lamari, 2002). As a result, a total of 12,900 (215 isolates × 3 replicates × 4 treatment [3 fungicide concentrations + 1 control] × 5 measurements) data points were used to evaluate mancozeb tolerance.

### 2.3 Data analysis

Growth rates were estimated with a logistic model (Aguayo, Elegbede, Husson, Saintonge, & Marcais, 2014) based on the sizes of individual colonies quantified at each time point over the six‐day inoculation period under each mancozeb concentration. The initial colony size at the point of inoculation (day one) was set to 0.2 cm^2^ (πr^2^ = 3.14 × 0.25^2^), and the capacity of colony growth (*K*) for the logistic model was set to 63.6 cm^2^ (πr^2^ = 3.14 × 4.5^2^). Mancozeb tolerance was measured by the relative growth rate (RGR) of isolates in the presence and in the absence of the fungicide (Brunner, Stefansson, Fountaine, Richina, & McDonald, 2016; Zhan et al., 2006). The frequency distribution of mancozeb tolerance in the 215 *A. alternata* isolates was determined using a binning approach and visualized by a histogram in which each bin was marked with the midpoint value of its lower and upper boundaries. Analysis of variance for mancozeb tolerance was performed using the general linear model procedure (GLM) implemented in SAS 9.4 (SAS Institute), and least significant difference (Kokalisburelle, Butler, & Rosskopf, 2013) was used to compare mancozeb tolerance among *A. alternata* populations sampled from different collection sites.

Genetic variation and population differentiation for SSR marker loci were estimated by gene diversity and *G*_ST_ (Nei, 1973) using Popgene 3.2 (Yeh, Yang, Boyle, Ye, & Xiyan, 1999). Phenotypic variance of RGR was calculated and partitioned into sources attributable to isolate (*I*, random effect), population (*P*, random effect), and fungicide concentration (*C*, fixed effect) using SAS GLM and VARCOMP programs (SAS 9.4, SAS Institute) according to the model:

(1) $$Y_{\text{ript}}=M\;+\;I\left(P\right)\;+\;C\;+\;P\;+\;I\left(P\right)\times C\;+\;P\times C\;+\;E_{\text{ripc}}$$

where *Y*_ripc_ refers to the mean RGR of replicate *r* for isolate *i* in population *p* at concentration *c*. *M*,* P*,* I*(*P*), *I*(*P*) × *C*,* P *× *C,* and *E*_ript_ refer to the overall population mean, genetic variance among populations, genetic variance within populations, variance due to the genotype × concentration interaction, responses of populations to dose effect, and the variance among replicates, respectively (Zhan & McDonald, 2011). Population differentiation (*Q*_ST_) of RGR was estimated in a way similar to the population differentiation of SSR marker loci (*G*_ST_) by calculating the proportion of total genetic variation attributable to among population variation using the following formula as described previously (Qin et al., 2016; Yang et al., 2016):

(2) $$Q_{\text{ST}}=\frac{\delta_{\text{AP}}^{2}\;+\;\delta_{P\;\times \;E}^{2}/n}{\delta_{\text{AP}}^{2}\;+\;\delta_{P\;\times \;E}^{2}/n\;+\;\delta_{\text{WP}}^{2}}$$

where $\delta_{\text{AP}}^{2}$, $\delta_{\text{WP}}^{2}$, $\delta_{P\times E}^{2}$, and *n* represent the variance among populations, variance within populations, variance in the population × concentration interaction, and the number of environments (concentrations), respectively (Zhan & McDonald, 2011; Zhan et al., 2005).

Heritability of RGR in a population was estimated by dividing genetic variance within populations with total phenotypic variance, and phenotypic plasticity of RGR was calculated by dividing the variance of isolate–concentration interaction by total phenotypic variance (Shaw, 1989; Tonsor, Elnaccash, & Scheiner, 2013). Statistical differences between the overall *G*_ST_ in SSR loci and overall *Q*_ST_ in fungicide sensitivity were evaluated using the standard deviation of *Q*_ST_ constructed from 100 resamplings of the original data (Zhan & McDonald, 2011).

Temperature and precipitation data for each collection site were downloaded from Weather Network (http://www.tianqi.com/). Annual mean and variation in temperature and precipitation at each collection site were estimated based on the weather data recorded over the last 10 years. Differences in annual mean temperature and precipitation between pairs of collection sites were estimated by dividing the absolute difference in annual mean temperature and precipitation with the sum of annual mean temperatures and precipitation in the two sites, respectively. UV radiation in a location is mainly affected by its vertical distance from sea level (Blumthaler, Ambach, & Ellinger, 1997) and was estimated by altitude in collection sites. The associations of mancozeb tolerance (population mean or *Q*_ST_) with local temperature, precipitation, and UV radiation (annual mean in each collection or pairwise difference in annual mean between collection site) and pairwise population differentiation in SSR marker loci (*G*_ST_) were evaluated by simple linear correlation (Lawrence & Lin, 1989) and second‐order polynomial correlation (Kniskern & Rausher, 2007).

## 3 RESULTS

### 3.1 Frequency distribution of mancozeb sensitivity in *Alternaria alternata* field populations

**Figure 2:**
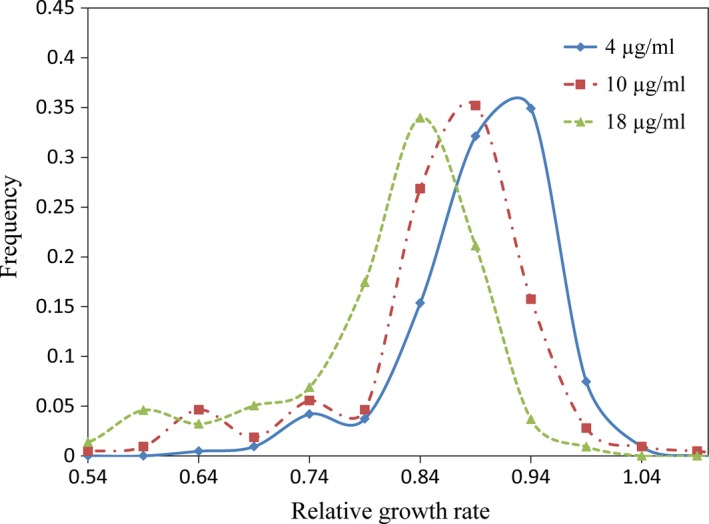
Frequency distribution of mancozeb tolerance at three concentrations in the 215 isolates of *Alternaria alternata* collected from seven potato fields across China. Mancozeb tolerance of isolates was measured with relative growth rate (RGR) of the isolates in the presence of mancozeb and in the absence of mancozeb

Between 28 and 32 genetically distinct isolates from each *A. alternata* population were assayed for mancozeb sensitivity in three concentrations by calculating the RGR of the pathogen in the presence and absence of the fungicide. RGR of the 215 isolates displayed a continuous and unimodal distribution in all three concentrations with a long tail stretching to low RGR (Figure 2), RGR ranging from 0.66 to 1.08 at the 4 μg/ml mancozeb treatment, 0.58 to 1.10 at the 10 μg/ml, and 0.45 to 1.01 at the 18 μg/ml, respectively. As fungicide concentration increased, the mean RGR of *A. alternata* populations decreased but ratio of RGR in the fastest and slowest growth isolates increased. In the 4 μg/ml mancozeb treatment, the ratio of RGR between the fastest and slowest growth isolates in the population was 1.638, but increased to 1.905 for the 10 μg/ml treatment and 2.262 for the 18 μg/ml treatment, respectively.

### 3.2 Genetic variation in SSR marker loci and mancozeb tolerance

The average SSR diversity in the seven *A. alternata* populations ranged from 0.31 to 0.62 with a grand mean of 0.41 (Table 1). The *A. alternata* population collected from FJN displayed the highest level of SSR diversity, while that collected from YNN displayed the lowest SSR diversity. Estimated heritability in the seven populations ranged from 0.22 to 0.51 with a mean of 0.36, while phenotypic plasticity in the seven populations ranged from 0.00 to 0.06 with a mean of 0.03. Heritability was 7‐ to 42‐fold times higher than phenotypic plasticity (average difference was 20‐fold) in the seven populations. There were no associations between gene diversity in SSR marker loci and heritability (*r* = −.34, *df* = 5, *p* = .46) or phenotypic plasticity (*r *=* *.03, *df* = 5, *p* = .95) in mancozeb tolerance.

**Table 1:** Sample size, annual mean temperature, gene diversity in SSR marker loci, and mean, heritability, and phenotypic plasticity of mancozeb tolerance in the seven *Alternaria alternata* populations from potato

				Mancozeb tolerance (RGR)			
Pop	Sample size	Annual mean temperature	Gene diversity	Mean	Heritability	Phenotypic plasticity	Heritability: Plasticity
HLJ	31	4.33	0.36	0.917A	0.22	0.00	‐
HNN	32	14.75	0.37	0.888B	0.37	0.04	9
FJN	28	20.54	0.62	0.884B	0.27	0.02	14
IMG	33	7.71	0.39	0.874C	0.42	0.01	42
SDG	30	14.83	0.40	0.873C	0.37	0.05	7
HBI	30	16.08	0.39	0.869C	0.51	0.06	9
YNN	31	15.63	0.31	0.859D	0.39	0.01	39
Mean	31	13.41	0.41	0.881	0.36	0.03	20

### 3.3 Differences in mancozeb tolerance among *Alternaria alternata* populations

Population, isolate, and mancozeb concentration all contributed significantly (*p* < .0001) to the variance in fungicide tolerance (Table 2). The 215 *A. alternata* isolates also responded differentially to different concentrations of mancozeb. The pathogen population from YNN showed the least tolerance to mancozeb, while the pathogen population from HLJ was the most tolerant to mancozeb (Table 1). The populations from FJN, HBI, and IMG all showed a medium level of mancozeb tolerance.

**Table 2:** Analysis of variance (ANOVA) of mancozeb tolerance in the 215 isolates of *Alternaria alternata* sampled from seven potato fields in China

Source	*df*	SS	Mean SS	*F* value	*p*
Population	6	1.47	0.246	39.84	<.0001
Concentration	2	5.42	2.711	439.83	<.0001
Isolate	211	22.79	0.108	17.53	<.0001
Concentration × Isolate	429	3.66	0.009	1.38	<.0001
Error	3957	24.39	0.006		

### 3.4 Spatial distribution of genetic variation in SSR marker loci and mancozeb tolerance

The overall population differentiation (*G*_ST_) in SSR across the seven pathogen populations was 0.12, which was significantly higher than 0.04, the overall population differentiation (*Q*_ST_) of mancozeb tolerance as measured by RGR. The pairwise *G*_ST_ in SSR marker loci was also higher than the pairwise *Q*_ST_ except for the comparison between HLJ and the other populations (Table 3). Pairwise *G*_ST_ in SSR marker loci was not associated with pairwise *Q*_ST_ in RGR (r_19_ = −0.13, *p* = .60).

**Table 3:** Pair‐population differentiation of SSR marker loci (*G*_ST_) and mancozeb tolerance (*Q*_ST_) among the seven populations of *Alternaria alternata* sampled from potato

	FJN	SDG	HBI	HNN	YNN	NMG	HLJ
FJN	‐	0.08	0.09	0.10	0.12	0.12	0.11
SDG	0.00	‐	0.01	0.02	0.03	0.04	0.03
HBI	0.02	0.00	‐	0.02	0.02	0.03	0.02
HNN	0.00	0.00	0.01	‐	0.04	0.03	0.02
YNN	0.02	0.00	0.00	0.02	‐	0.04	0.02
IMG	0.00	0.00	0.01	0.00	0.01	‐	0.10
HLJ	0.18	0.16	0.25	0.13	0.28	0.11	‐

Values above the diagonal are *G*_ST_, and values below the diagonal are *Q*_ST._

### 3.5 Associations of site temperature, precipitation, and UV radiation with mancozeb tolerance

**Figure 3:**
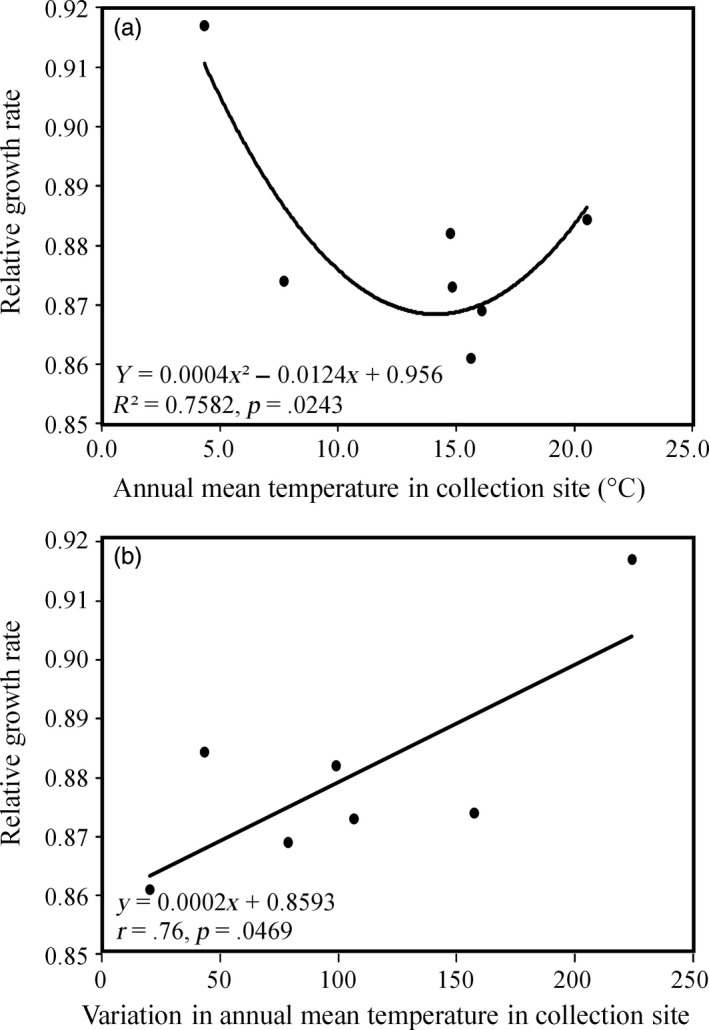
Correlation between the mean mancozeb tolerance in *Alternaria alternata* populations and annual mean temperature and variation in temperature in the collection sites: (a) annual mean temperature in the collection sites, (b) variation in temperature in the collection sites. Mancozeb tolerance of populations was measured with mean relative growth rate (RGR) of isolates in the presence of mancozeb and in the absence of mancozeb across three fungicide concentrations. Variance of annual temperature at collection sites was estimated based on the mean air temperature across 12 months

**Figure 4:**
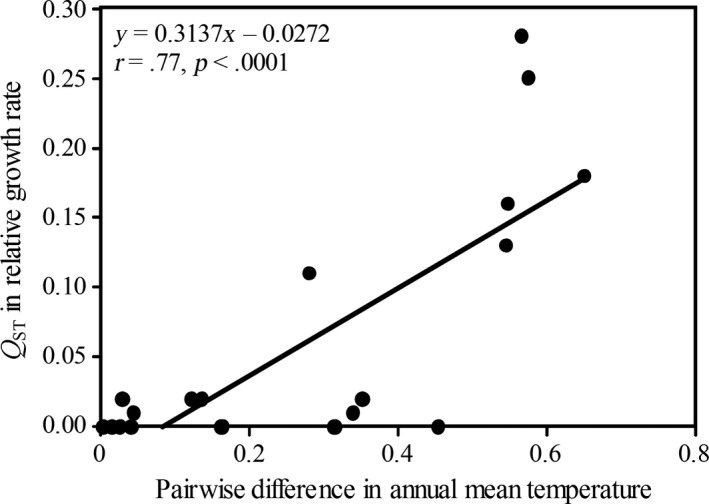
Correlation between the pairwise genetic differentiation (*Q*_ST_) of mancozeb tolerance in *Alternaria alternata* populations and the pairwise difference in annual mean temperature in the collection sites. Mancozeb tolerance of isolates was measured with relative growth rate (RGR) of the isolates in the presence of the fungicide and in the absence of the fungicide. Pairwise difference in annual mean temperature was estimated by dividing the absolute difference in annual mean temperature in two collection sites with the sum of annual mean temperature in the two sites

Mean mancozeb tolerance in *A. alternata* populations was significantly correlated with the annual mean and variation in temperature in the collection sites (Figure 3). Overall, mean mancozeb tolerance of *A. alternata* populations in nature followed a U‐shape distribution across the thermal gradient represented by the collection sites, initially decreasing along with annual mean temperature, reaching a minimum at ~15°C of local annual mean temperature and then increasing again at higher local mean temperatures (Figure 3a). The pairwise population differentiation in mancozeb tolerance (*Q*_ST_) was positively correlated (*r *=* *.77, *df* = 19, *p* < .0001) with the pairwise difference in annual mean temperature between collection sites (Figure 4). Mancozeb tolerance was not associated with local precipitation and UV radiation (Figs. S1 and S2).

## 4 DISCUSSION

In this study, we examined the interaction between local climatic factors and evolution of fungicide resistance, an important issue that has attracted considerable public interests but has rarely been considered together. Our study centered on a population genetic survey of tolerance to a nonspecific fungicide in plant pathogen populations sampled from a range of geographic locations varying in thermal conditions and ecotypes across China (Figure 1). Our results confirm the low risk of developing mancozeb resistance in natural plant pathogen populations (Gullino et al., 2010). Due to the large number of isolates assayed, we only tested the sensitivity of the pathogen at three mancozeb concentrations. Although this limitation does not allow us to calculate EC_50_, the half maximum effective concentration, accurately (Sebaugh, 2011), a rough estimate indicates that it ranges from 0.02 to 39.57 μg/ml with a mean of 12.79 μg/ml, which was within the ranges observed for *A. alternata* in other studies (Malandrakis et al., 2015) as well as for other pathogens (Elliott, Shamoun, & Sumampong, 2015; Rekanovic et al., 2012).

Three factors may contribute individually or jointly to the low risk of developing resistance to nonspecific fungicides such as mancozeb in natural populations of plant pathogens. Mutations for mancozeb resistance may be low due to the nonspecific and multisite action of the fungicide. However, low mutation seems to be a less plausible explanation in this case as a rapid increase in mancozeb tolerance has been observed in an animal system under laboratory conditions. After 10 generations of artificial selection, 50% lethal concentration (LC_50_) of mancozeb in *Typhlodromus pyri,* a predatory mite found in many fruit‐growing regions of the world, increased more than 70 times (Auger, Bonafos, Kreiter, & Delorme, 2005), and it is expected that fungal pathogens can adapt faster due to their generally escalated evolutionary rates relative to animals (Neff et al., 2005).

*Alternaria alternata* can infect a wide range of host plants including many wild species (Wier et al., 1998; Woudenberg et al., 2013) which are usually not exposed to synthetic fungicides such as mancozeb, therefore favoring the conservation of highly sensitive genotypes in the pathogen populations. It is likely that *A. alternata* in some of these wild plant species also act as a source of primary inoculum leading to early blight disease of potatoes in China and continuous influx of the highly sensitive genotypes from the pathogen populations reserved in wild host species prevents or substantially reduces the risk of developing fungicide resistance such as mancozeb in agriculture. Negative pleiotropy between fungicide resistance and ecological adaptation of pathogens to biotic and other abiotic environments can also lead to a low evolutionary risk of developing resistance to nonspecific antimicrobials such as mancozeb in nature as documented in other studies (Darmency, Menchari, Le Corre, & Delye, 2015; MacLean, Hall, Perron, & Buckling, 2010). Negative pleiotropy can generate differential impact on the evolution of antimicrobial resistance and local population dynamics to those caused by fitness penalty. Under a fitness penalty, resistant mutants have a selective advantage in the presence of antimicrobials but display a reduced competitive ability in the absence of the corresponding antimicrobials due to modification of vital cellular functions (Markoglou, Malandrakis, Vitoratos, & Ziogas, 2006). This can result in significant levels of spatiotemporal variation in mean resistance and resistance frequency in natural pathogen populations (Boerlin et al., 2005; Courvalin, 2005). Trade‐offs resulting from negative pleiotropy with other ecological and life‐history traits of pathogens may counterbalance the selective advantages of resistant mutations even in the presence of the antimicrobials, hampering accumulation and spatiotemporal dynamics of mutants. This trade‐off is expected to be common (Hall, Gubbins, & Gilligan, 2004; Li & Xiao, 2008), particularly in nonspecific fungicides (Melnyk, Wong, & Kassen, 2015) such as mancozeb, due to the involvement of many genes across pathogen genomes in the evolution of resistance and may also explain the constraining selection in the adaptation of *A. alternata* to mancozeb as indicated by a significantly lower population differentiation in mancozeb tolerance than that of SSR marker loci as well as similar spatiotemporal patterns of mancozeb sensitivity observed across pathogen species (Elliott et al., 2015; Henríquez, Sarmiento, & Alarcón 2011).

Climatic factors such as local temperature, precipitation, and UV irradiation may exert influence on many aspects of biological, ecological, and evolutionary processes of species (Knies & Kingsolver, 2010; Siepielski et al., 2017; Singaravelan et al., 2008; Zhan & McDonald, 2011). They could interact with pathogens to shape the evolutionary trajectory of antimicrobial adaptation. When we analyzed the associations of these climatic factors with antimicrobial parameters in the current study*,* we found a significant correlation between the pairwise genetic differentiation in mancozeb tolerance (*Q*_ST_) in *A. alternata* populations and the pairwise differences in annual mean temperature between the sites where the pathogen populations were collected (Figure 4). We also found that the mean mancozeb tolerance of *A. alternata* population was significantly correlated with the annual mean and variation in local temperature in the collection sites (Figure 3). No such associations were found between mancozeb tolerance of *A. alternata* populations with local precipitation and UV radiation (Figs. S1 and S2). The observed association between mancozeb tolerance and local thermal condition is unlikely caused by sample bias because the pathogen populations were randomly selected from our national collections and there was not association between geographic vicinity (Figure 1) and tolerance similarity (Table 2) of the pathogen. These results suggest that temperature is the main climatic factor which can strongly impact the evolution of antimicrobial resistance, possibly resulting from the trade‐off of antimicrobial adaptation with thermal adaptation in pathogens. Further analysis indicates that mancozeb tolerance in *A. alternata* was convexly correlated with local mean temperature: reduced first to a threshold as local mean temperature increased but bounced back as local mean temperature increased further (Figure 3a). This pattern is consistent with previous reports showing that temperature modifies the amplitude of fitness in antimicrobial resistance or even shifts the effects of resistant mutants from benefits to damages (Rodríguez‐Verdugo et al., 2013; Zhang, Yang, & Pruden, 2015). For example, mutations to rifampicin resistance in the *rpoB* gene of *Escherichia coli* were selectively advantageous under a high temperature coupled with nutrient limitation but displayed a fitness cost under a low thermal condition (Rodríguez‐Verdugo et al., 2013).

Like other phenotypic traits, antimicrobial resistance in pathogen individuals is determined primarily by inherited genes (heritability) and their differential expressions (plasticity, Mohd‐Assaad et al., 2016). Plasticity whereby a genotype produces different phenotypes through changes in gene expression is an important feature of species to adapt the constant fluctuation of environmental factors such as daily variation in air temperature (Chen, Nolte, & Schlötterer, 2015; Yang et al., 2016). In the current study, heritability accounts for a third of phenotypic variation, while plasticity is negligible (Table 1), suggesting that genetic variance in allelic composition plays a more important role in the adaptation of *A. alternata* to mancozeb than regulation of gene expression by environmental exposure. Lack of plasticity may also contribute to the negative association between mancozeb tolerance and variation in local temperature. This contradicts our previous work on the adaptation of *Phytophthora infestans* to azoxystrobin (Qin et al., 2016), which was also focused on these potato‐growing regions in China. This inconsistency could result from the different action modes in azoxystrobin and mancozeb. In contrast to mancozeb, azoxystrobin is a site‐specific fungicide (Gisi & Sierotzki, 2008). Site‐specific fungicides such as azoxystrobin select for polarized genotypes that are either sensitive or highly resistant, leading to considerable variation (plasticity) of pathogens to changing environments. On the other hand, broad‐spectrum fungicides such as mancozeb select for similar genotypes, which only vary quantitatively in their level of sensitivity (tolerance), maximizing the contribution of genetic effect (heritability) but minimizing the particular interaction between genotype and environment (plasticity). This variation in action modes between the two fungicides may also contribute to the distinct patterns of association between local temperature and fungicide tolerance. A linear negative correlation between annual mean temperature and azoxystrobin tolerance in *P. infestans* was found in the study (Qin et al., 2016) as compared to the convex correlation observed here.

Rather than mean temperature in growing season, we used annual mean temperature to analyze the interaction between mancozeb and thermal adaptation as performed by previous studies (Stefansson, Willi, Croll, & McDonald, 2014; Wu et al., 2016) for two reasons. First, adaptation of pathogens to thermal and other environmental conditions occurs over multiple years, not only during the epidemic phase of the growing seasons when hosts are available, but also during the saprotrophic phase of off‐seasons when hosts are absent and pathogens usually display a differential adaptation during the two phases of life cycle (Abang et al., 2006; Sommerhalder, McDonald, Mascher, & Zhan, 2010). Second, the length of the growing season is difficult to determine for many pathogens with multiple hosts (such as *A. alternata*) due to cross‐species transmission (Dobson, 2004; Woolhouse, 2001). Regardless, the same associations were found when the mean temperature of potato‐growing season in collection sites was used although significance levels were reduced (data not shown).

Human activity may drive the increase and fluctuation of global temperature substantially (IPCC 2007). There is wide concern that such global trends of temperature change may have major impacts on food production and public wealth through their influence on the continued efficacy and sustainability of the antimicrobials used to treat human, animal, and plant diseases. The finding of a negative association between thermal fluctuation and population mean tolerance (Figure 3b) suggests that increase in temperature fluctuation associated with future climate change may reduce the efficacy of mancozeb and other antimicrobials with similar chemical features and action modes, signaling a threat to public and animal health, food security, and ecological sustainability, particularly in tropical and subtropical regions. This result is alarming but further investigation in a broad context with a sophisticated tool such as experimental evolution approach (e.g., Yang et al., 2016) is required to confirm the finding.

## AUTHOR CONTRIBUTIONS

MHH collected pathogen isolates, generated and analyzed the data, and wrote the manuscript; DLL, WZ, EJW, LNY, and WA collected pathogen isolates and generated the data; and JZ conceived and designed the experiments, analyzed the data, and wrote the manuscript.

## DATA ACCESSIBILITY

Data available from the Dryad Digital Repository: https://doi.org/10.5061/dryad.bp81r
