# Supplementary material for: Slow and temperature‐mediated pathogen adaptation to a nonspecific fungicide in agricultural ecosystem
Source: Evol Appl. 2017 Sep 14;11(2):182–92. doi: 10.1111/eva.12526 (PMC5775493; doi:10.1111/eva.12526)
Supplement: Supplementary file 1 [file EVA-11-182-s001.docx]

Supporting Information for:

**Slow and temperature-mediated pathogen adaptation to a non-specific fungicide in agricultural ecosystem**


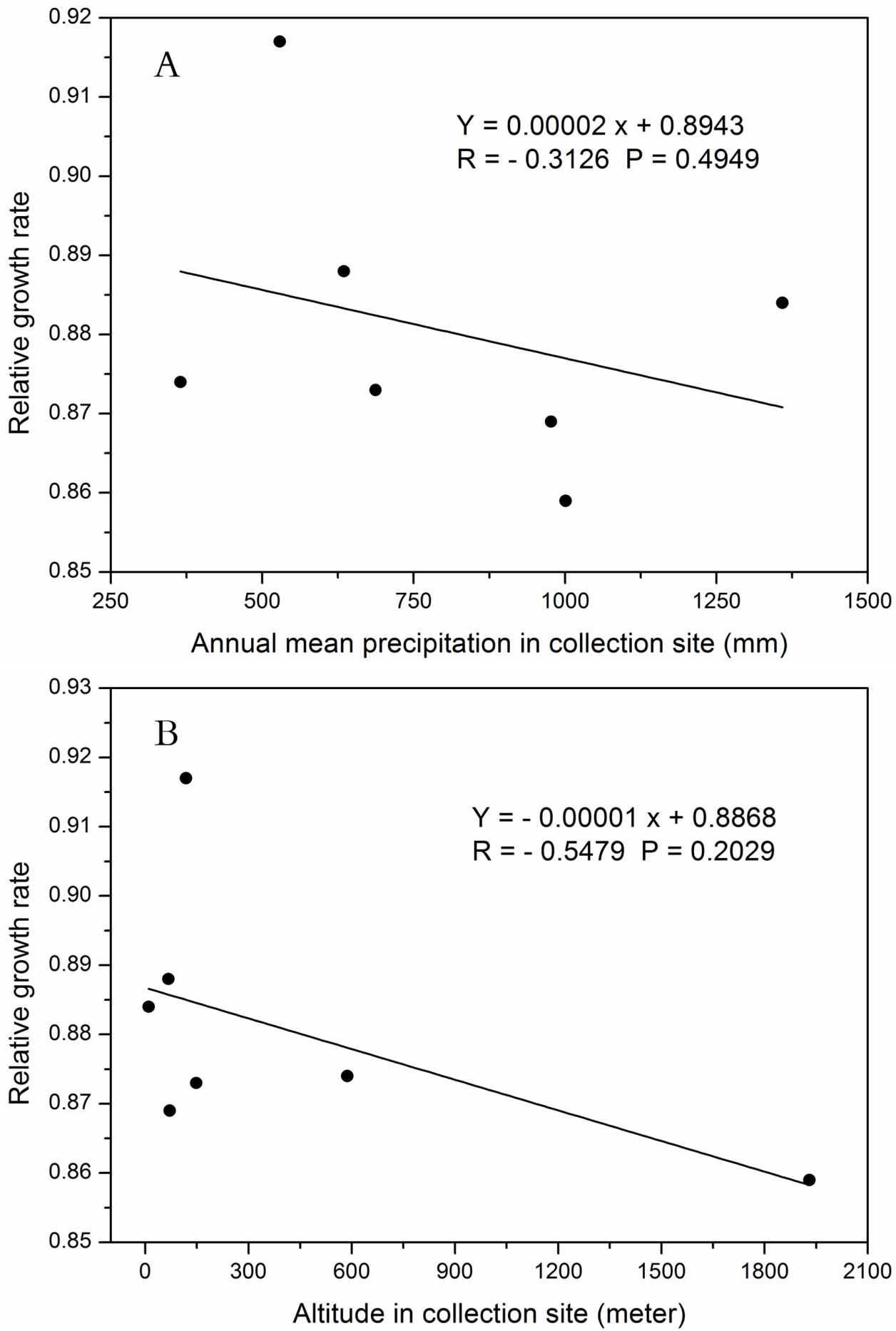


Figure S1 Correlation of the mean mancozeb tolerance in *Alternaria alternata* populations with annual mean precipitation and UV radiation in the collection sites. Mancozeb tolerance of populations was measured with mean relative growth rate (RGR) of isolates in the presence of mancozeb to the absence of mancozeb cross three fungicide concentrations. UV radiation was inferred from altitude in collection sites: (A) annual mean precipitation in the collection sites, (B) UV radiation as indicated by altitude in the collection sites.


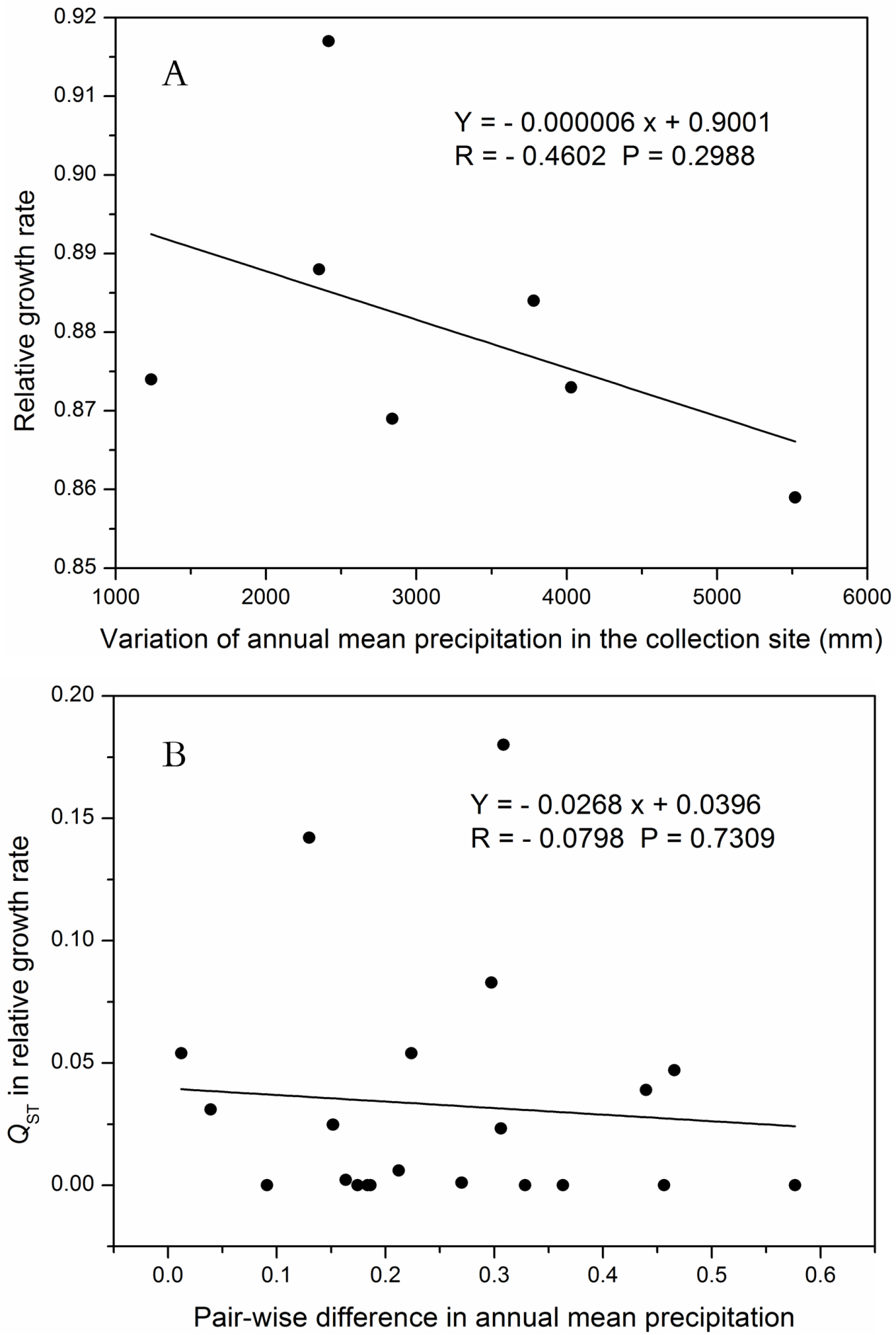


Figure S2 Correlation between the mancozeb tolerance in *Alternaria alternata* populations and precipitation in the collection sites: (A) mean mancozeb tolerance in *A*. *alternata* populations and variation of annual mean precipitation in the collection sites, (B) pairwise genetic differentiation (*Q*_ST_) of mancozeb tolerance in *A*. *alternata* populations and the pairwise difference of annual mean precipitation in the collection sites. Mancozeb tolerance of populations was measured by mean relative growth rate (RGR) of isolates in the presence of mancozeb to the absence of mancozeb cross three fungicide concentrations. Variance of annual precipitation at collection sites was estimated based on the monthly precipitation across 12 months in a year. Pairwise difference of annual mean precipitation was estimated by dividing the absolute difference of annual mean precipitation in two collection sites by the sum of annual mean precipitation in the two sites.
